# Mapping the genetic–transcriptional landscape of thyroid irAEs in sintilimab therapy: toward biomarker-guided immunotoxicity prediction

**DOI:** 10.3389/fimmu.2025.1671594

**Published:** 2025-10-27

**Authors:** Wei Chen, Mingyu Zhang, Taifeng Li, Bing Shang, Haishuai Su, Yafei Shi, Yutao Liu, Feng Yu, Guohui Li

**Affiliations:** ^1^ School of Basic Medicine and Clinical Pharmacy, China Pharmaceutical University, Nanjing, Jiangsu, China; ^2^ Department of Pharmacy, National Cancer Center/National Clinical Research Center for Cancer/Cancer Hospital, Chinese Academy of Medical Sciences and Peking Union Medical College, Beijing, China; ^3^ Department of Medical Oncology, National Cancer Center/National Clinical Research Center for Cancer/Cancer Hospital, Chinese Academy of Medical Sciences and Peking Union Medical College, Beijing, China

**Keywords:** immune-related adverse events (irAEs), sintilimab, eQTL analysis, complement system, longitudinal transcriptomics

## Abstract

**Objective:**

By integrating whole-genome resequencing (WGR) with longitudinal transcriptomic profiling, this study aimed to unravel the genetic–transcriptional regulatory network underlying thyroid immune-related adverse events (irAEs) in non-small cell lung cancer (NSCLC) patients treated with sintilimab. A key objective was to identify molecular biomarkers with predictive and therapeutic relevance.

**Methods:**

This prospective study included NSCLC patients receiving sintilimab, from whom peripheral blood samples were collected at three time points: baseline, post-first treatment, and post-second treatment. RNA sequencing (RNA-seq) and 30× WGR were performed. Differential gene expression analysis was conducted on the RNA-seq data, followed by longitudinal consistency filtering using the Longitudinal Concordant Gene Intersection (LCGI) algorithm to identify robust differentially expressed genes (DEGs). These DEGs underwent downstream integration with protein–protein interaction (PPI) network analysis and cis-expression quantitative trait loci (cis-eQTL) mapping to pinpoint key genes and regulatory single-nucleotide polymorphisms (SNPs) associated with thyroid irAEs.

**Results:**

The LCGI algorithm identified 13 DEGs exhibiting sustained directional shifts across treatment timepoints. Integration with conventional DEG signatures revealed a functionally cohesive module, with C1QA/B/C, FLT1, TEK, PDGFRB, SPP1, and HLA-DPB1 emerging as central regulators of thyroid irAEs. Cis-eQTL mapping identified 500 SNPs with significant cis-regulatory effects on 153 genes. A “C3 complement-matrix axis” was uncovered as a pivotal node, promoting macrophage polarization toward a pro-inflammatory phenotype. Based on the refined PPI network, we proposed a cascading pathological model in which a self-sustaining feedback loop drove progressive and irreversible thyroid autoimmunity.

**Conclusion:**

This study established a genetic–transcriptional regulatory framework for sintilimab-induced thyroid irAEs and identified a candidate gene set with biomarker potential. Our findings highlighted the central role of complement-driven mechanisms, providing a foundation for precision risk prediction and targeted intervention strategies that preserve antitumor efficacy while mitigating autoimmune toxicity.

## Introduction

Immune checkpoint inhibitors (ICIs) have revolutionized the therapeutic landscape of non-small cell lung cancer (NSCLC), offering durable clinical benefit in a subset of patients (1, 2). Sintilimab, a fully human IgG4 monoclonal antibody targeting PD-1, exhibits high-affinity binding and sustained blockade of the PD-1/PD-L1 pathway (3), leading to significantly improved survival outcomes in NSCLC (4). However, thyroid immune-related adverse events (irAEs) have emerged as some of the most prevalent toxicities associated with ICI-based regimens, with incidence rates approaching 50% in combination therapies (5). These endocrine irAEs span a clinical spectrum that includes hypothyroidism, hyperthyroidism, and destructive thyroiditis, often necessitating treatment discontinuation and substantially impairing patient quality of life (6, 7). Despite its proven clinical efficacy, the genetic and transcriptomic underpinnings of sintilimab-induced thyroid irAEs in NSCLC remain largely undefined.

The advancement of gene sequencing technologies has enabled major breakthroughs in elucidating the genetic basis of disease pathogenesis (8, 9). Genome-wide association studies (GWAS) have been widely used to identify genetic loci associated with various traits and diseases, but they generally require large cohorts to achieve statistically robust and reproducible genome-wide associations (10). As a result, for studies involving small sample sizes, such as investigations into adverse drug reactions, GWAS often prove insufficient as a stand-alone analytical strategy. Moreover, even in large-scale GWAS, although numerous trait-associated loci have been successfully identified, the mechanistic interpretation of these variants remains an ongoing challenge: the specific molecular pathways through which these variants influence biological functions and disease phenotypes are often poorly understood (11).

Expression quantitative trait loci (eQTL) studies illuminate the functional consequences of genetic variants by linking them to transcriptional changes, offering critical mechanistic insights into GWAS-identified loci and the etiology of complex diseases (12). The integration of multiple expression modalities, such as total gene expression and allele-specific expression, significantly enhances both the sensitivity and specificity of eQTL detection, particularly in heterogeneous tissues like tumors. Advanced mapping strategies such as pTReCASE, which incorporate tumor purity estimates and distinguish between tumor-derived and normal cellular expression, offer a more refined and accurate framework for detecting tumor-specific regulatory effects (13). These methodological advances not only improve the identification of disease-associated genes and actionable therapeutic targets but also deepen our understanding of the molecular circuitry driving disease progression (14). In the context of immunological research, eQTL analyses hold distinct advantages by capturing cell- and tissue-specific regulation, temporal expression dynamics, and multicellular interactions, thus providing powerful tools to dissect the genetic basis of immune-related disorders and inform novel therapeutic strategies (15).

Previous investigations into irAEs have largely been restricted to static comparisons of gene expression between patient groups following a single ICI administration. However, immune responses are inherently dynamic and temporally regulated. To better capture this complexity, the current study assessed transcriptomic profiles at three sequential time points: pre-treatment, post-first treatment, and post-second treatment. Differential expression and pathway enrichment analyses were performed to systematically characterize the evolving transcriptomic landscape in response to sintilimab administration.

This study leveraged transcriptomic data to perform an unbiased, genome-wide eQTL analysis aimed at uncovering single-nucleotide polymorphisms (SNPs) linked to gene expression changes implicated in thyroid irAEs. By integrating SNP profiles with longitudinal transcriptomic data, we reconstructed the genetic-transcriptional regulatory network underlying thyroid irAEs, thus addressing a key limitation in this field, namely, the constraints of small sample sizes in rare adverse event studies. This multi-omics approach illuminated the mechanistic connections between genetic variation and the emergence of thyroid irAEs, revealing candidate molecular pathways that might drive their development. Our findings also pointed to a set of clinically actionable biomarkers with potential utility for dynamic risk stratification in NSCLC patients undergoing sintilimab treatment. Through this comprehensive framework, we provided new insights into the genetic architecture of immune toxicity, with broader implications for precision medicine in cancer immunotherapy.

## Materials and methods

### Study design and participants

This study was designed as a prospective, real-world investigation. Eligible participants met the following criteria: (1) hospitalization at the National Cancer Center/Cancer Hospital, Chinese Academy of Medical Sciences between January 2021 and December 2024; (2) histological or clinical confirmation of NSCLC; (3) completion of at least two cycles of sintilimab administered every 3 weeks; (4) age between 18 and 80 years; and (5) availability of complete and evaluable baseline (BT) data prior to treatment initiation.

Exclusion criteria included: (1) history of thyroid surgery or radiotherapy to the cervical region; (2) preexisting thyroid disease or dysfunction; and (3) severe cardiac, hepatic, renal, or autoimmune disorders.

The study protocol was approved by the Ethics Committee of the National Cancer Center/Cancer Hospital, Chinese Academy of Medical Sciences (approval number: 2020122509100302). Written informed consent was obtained from all participants before enrollment.

### Whole-Genome Resequencing

Peripheral blood samples were collected, and genomic DNA was isolated using the TIANamp Genomic DNA Kit. DNA concentration was quantified with a Qubit^®^ 3.0 Fluorometer (Life Technologies, CA, USA), while integrity and purity were assessed via 1% agarose gel electrophoresis (120 V, 45 min). Library preparation was performed following the TruSeq DNA Sample Preparation Guide (Illumina, 15026486 Rev. C). Library quality was verified by qPCR using the Bio-Rad iQ SYBR Green Kit.

Sequencing was conducted on the Illumina NovaSeq 6000 platform using paired-end (PE) protocols, generating 150-bp PE reads with an average coverage depth of 30×. Clean reads were aligned to the UCSC *hg38* human reference genome using BWA. Variant calling was subsequently performed using the Genome Analysis Toolkit (GATK) to extract candidate polymorphic SNP loci.

### RNA sequencing

High-quality total RNA was extracted from peripheral blood samples. RNA purity was assessed using a NanoDrop spectrophotometer (NanoDrop Technologies, Wilmington, DE, USA), and RNA integrity was evaluated using the Agilent 2100/5400 Bioanalyzer (Agilent Technologies, Santa Clara, CA, USA). Ribosomal RNA was depleted from total RNA prior to library construction.

Fragmented RNA was used as a template for first-strand cDNA synthesis primed with random hexamers. Second-strand synthesis was performed using DNA Polymerase I, RNase H, and a dNTP mix in which dUTP replaced dTTP. The resulting double-stranded cDNA underwent purification, end repair, adenylation, and adapter ligation. Uracil-containing second strands were selectively digested with USER enzyme (New England Biolabs) to ensure strand specificity. Final libraries were purified using AMPure XP beads (Beckman Coulter). Libraries that passed quality control were sequenced on the Illumina NovaSeq X Plus platform using a 150-bp PE protocol.

### Statistical analyses

**Figure 1:**
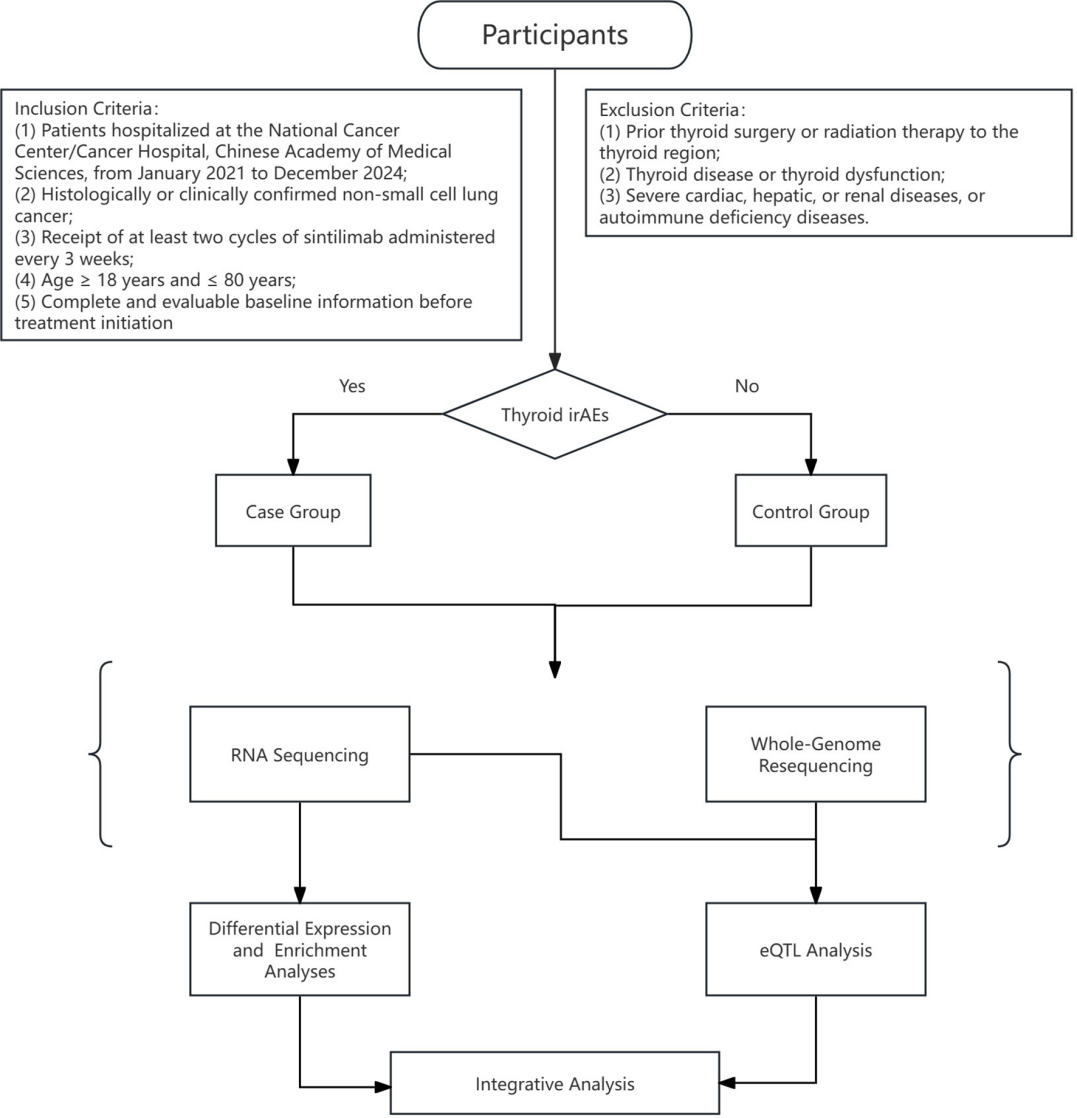
Research process for investigating immune-related thyroid dysfunction in NSCLC patients receiving sintilimab treatment.

The overall analytical workflow is illustrated in **Figure 1**. Differential gene expression analysis was conducted using the DESeq2 package (version 1.34.0), while eQTL mapping was performed using the Matrix eQTL package (version 2.3) in R (version 4.3.3).

### Differential expression analyses

RNA-seq quantification data were analyzed using the DESeq2 package (version 1.34.0) in R (version 4.3.3). Raw gene counts were imported from a preprocessed expression matrix, accompanied by metadata specifying experimental groups and covariates (sex and age). Genes with low expression, defined as fewer than 10 counts in fewer than *k* samples, where *k* corresponds to the sample size of the smallest comparison group, were excluded to reduce noise and retain biologically meaningful signals.

Categorical variables (e.g., sex and group) were encoded as factors, while continuous variables (e.g., age) were standardized via z-score transformation (mean-centered and scaled to unit variance). Differential expression testing was performed using a negative binomial generalized linear model that accounted for sex and age as covariates. Dispersion estimates were moderated using empirical Bayes shrinkage prior to Wald significance testing.

Differentially expressed genes (DEGs) were defined based on dual criteria: an absolute log_2_ fold change >1 and an adjusted *p*-value (padj, Benjamini–Hochberg correction) <0.05.

Gene expression can be influenced by a variety of environmental and biological factors, including diet and lifestyle (16), chemical exposure (17), hormonal fluctuations (18, 19), and cellular states such as proliferation and differentiation (20). To mitigate confounding from such variables, we implemented the Longitudinal Concordant Gene Intersection (LCGI) method, a robust analytic strategy designed to reduce false positives while prioritizing sustained biological signals. This approach incorporates longitudinal RNA-seq data from two post-treatment time points, filtering genes by fold-change thresholds (A1T >1, A2T >0.5), *p*-value significance (<0.05), and directional consistency across time. The final DEG set was defined as the intersection of genes meeting all criteria, thereby enriching for genes with persistent, treatment-associated expression changes.

### Gene ontology functional annotation and kyoto encyclopedia of genes and genomes pathway enrichment analysis

To minimize baseline confounding and achieve a comprehensive characterization of pathways associated with thyroid irAEs, we integrated enrichment analyses from two gene sets: (1) concordantly regulated DEGs identified via the LCGI method; and (2) combined DEGs from the post-treatment stages A1T and A2T, excluding those differentially expressed at BT.

GO functional annotation and KEGG pathway enrichment analyses were performed using the DAVID online platform (https://davidbioinformatics.nih.gov/home.jsp). Visualization of enrichment results was achieved through Sankey diagrams generated with Bioladder (https://www.bioladder.cn) and bubble plots created via Wei Sheng Xin (http://www.bioinformatics.com.cn).

### Construction of protein-protein interaction networks

DEGs across the three time points were compared and visualized using Venn diagrams. PPI networks were constructed based on these DEGs using the STRING database. The resultant interaction data were imported into Cytoscape software to analyze network topology and facilitate visualization. Key genes within the network were identified using the CytoHubba plugin employing the maximal clique centrality (MCC) algorithm, while functionally relevant gene clusters were detected using the MCODE plugin. Integration of these analyses enabled the identification of significantly connected and functionally important gene modules.

### eQTL analysis

Cis-acting genetic associations between SNPs and gene expression levels were identified using a linear model framework implemented in the MatrixEQTL R package (version 2.3). SNP-gene pairs located within a 1-Mb window flanking each gene’s transcription start site were tested for association. All significant cis-eQTLs were defined by a false discovery rate (FDR) threshold of <0.05 and retained for subsequent functional annotation.

### Integrative analysis

Genes identified through cis-eQTL mapping were intersected with DEGs derived from transcriptomic analyses. The overlapping gene sets were then analyzed via the STRING online platform, applying a network interaction confidence cutoff set to “confidence” to delineate high-confidence PPIs. Integrating these data with prior knowledge from the literature enabled the inference of putative molecular mechanisms driving thyroid irAEs.

## Results

### Patient characteristics

A total of 29 patients were enrolled in this study. Within this cohort receiving combined sintilimab and chemotherapy, 21 patients (72.41%) maintained normal thyroid function indices at BT, including T3, T4, FT3, FT4, and TSH, and exhibited persistently normal TSH levels following treatment. In contrast, eight patients (27.59%) with normal BT thyroid function developed abnormal TSH levels post-treatment (**Table 1**). Importantly, no statistically significant differences were observed between these two groups with respect to sex, age, tumor histological subtype, disease stage, concomitant medication regimens, or BT thyroid function parameters.

**Table 1:** BT characteristics and clinical parameters comparison between patients with normal thyroid function and thyroid dysfunction after sintilimab combined with chemotherapy treatment.

Characteristic	Normal Thyroid Function (n=21) N = 21^1^	Thyroid Dysfunction (n=8) N = 8^1^	p-value^2^
Age			0.59
Mean (SD)	61 (10)	64 (10)	
Median (Q1, Q3)	61 (56, 68)	67 (55, 72)	
Min, Max	41, 77	50, 77	
Sex			>0.99
female	8 (38%)	3 (38%)	
male	13 (62%)	5 (63%)	
Histological Type			0.65
adenocarcinoma	16 (76%)	5 (63%)	
squamous carcinoma	5 (24%)	3 (38%)	
Thyroid-stimulating hormone			0.17
Mean (SD)	2.34 (1.06)	1.99 (1.68)	
Median (Q1, Q3)	2.26 (1.54, 2.79)	1.59 (0.84, 2.32)	
Min, Max	0.72, 5.63	0.66, 5.82	
Staging			0.28
II	0 (0%)	1 (13%)	
III	4 (19%)	3 (38%)	
IV	16 (76%)	4 (50%)	
missing	1 (4.8%)	0 (0%)	
T stage			0.59
T1	2 (9.5%)	2 (25%)	
T2	4 (19%)	3 (38%)	
T3	4 (19%)	1 (13%)	
T4	10 (48%)	2 (25%)	
X	1 (4.8%)	0 (0%)	
N stage			0.15
N0	3 (14%)	1 (13%)	
N1	1 (4.8%)	0 (0%)	
N2	5 (24%)	5 (63%)	
N3	11 (52%)	1 (13%)	
X	1 (4.8%)	1 (13%)	
M stage			0.14
M0	3 (14%)	4 (50%)	
M1	15 (71%)	4 (50%)	
X	3 (14%)	0 (0%)	
Non-platinum			0.32
None	0 (0%)	1 (13%)	
Pemetrexed	11 (52%)	4 (50%)	
Taxanes	9 (43%)	2 (25%)	
Vinorelbine	1 (4.8%)	1 (13%)	
Platinum-based			0.43
Carboplatin	10 (48%)	4 (50%)	
Cisplatin	0 (0%)	1 (13%)	
None	11 (52%)	3 (38%)	
Bevacizumab			>0.99
Bevacizumab	9 (43%)	3 (38%)	
None	12 (57%)	5 (63%)	

^1^n (%).

^2^Wilcoxon rank sum test; Fisher’s exact test; Wilcoxon rank sum exact test.

### Differential expression analyses and dynamic expression patterns

**Figure 2:**
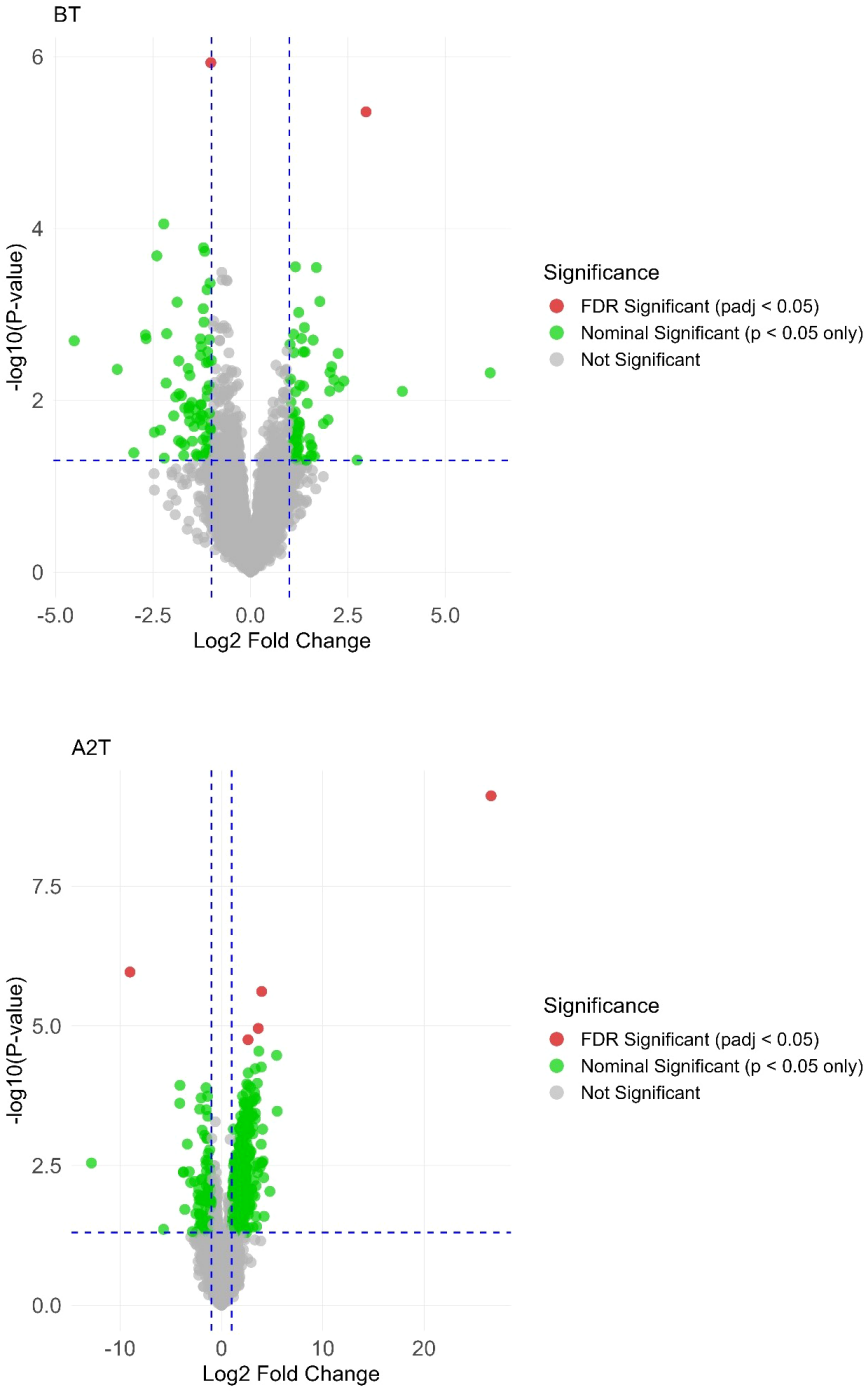
Volcano plot highlighting significant gene expression variations across the 3 distinct stages: BT, A1T, A2T.

Differential expression analysis was performed to identify genes exhibiting significant expression differences between the thyroid irAEs group and controls across three distinct timepoints (BT, A1T, A2T). Volcano plots (**Figure 2**) illustrate gene expression changes at each stage. Raw *p*-values were adjusted using the Benjamini–Hochberg procedure to control the FDR, generating padj. In these plots, red dots denote genes meeting the criteria of padj < 0.05 and absolute log_2_ fold change > 1, while green dots indicate genes with padj < 0.05 and absolute log_2_ fold change > 1.

**Figure 3:**
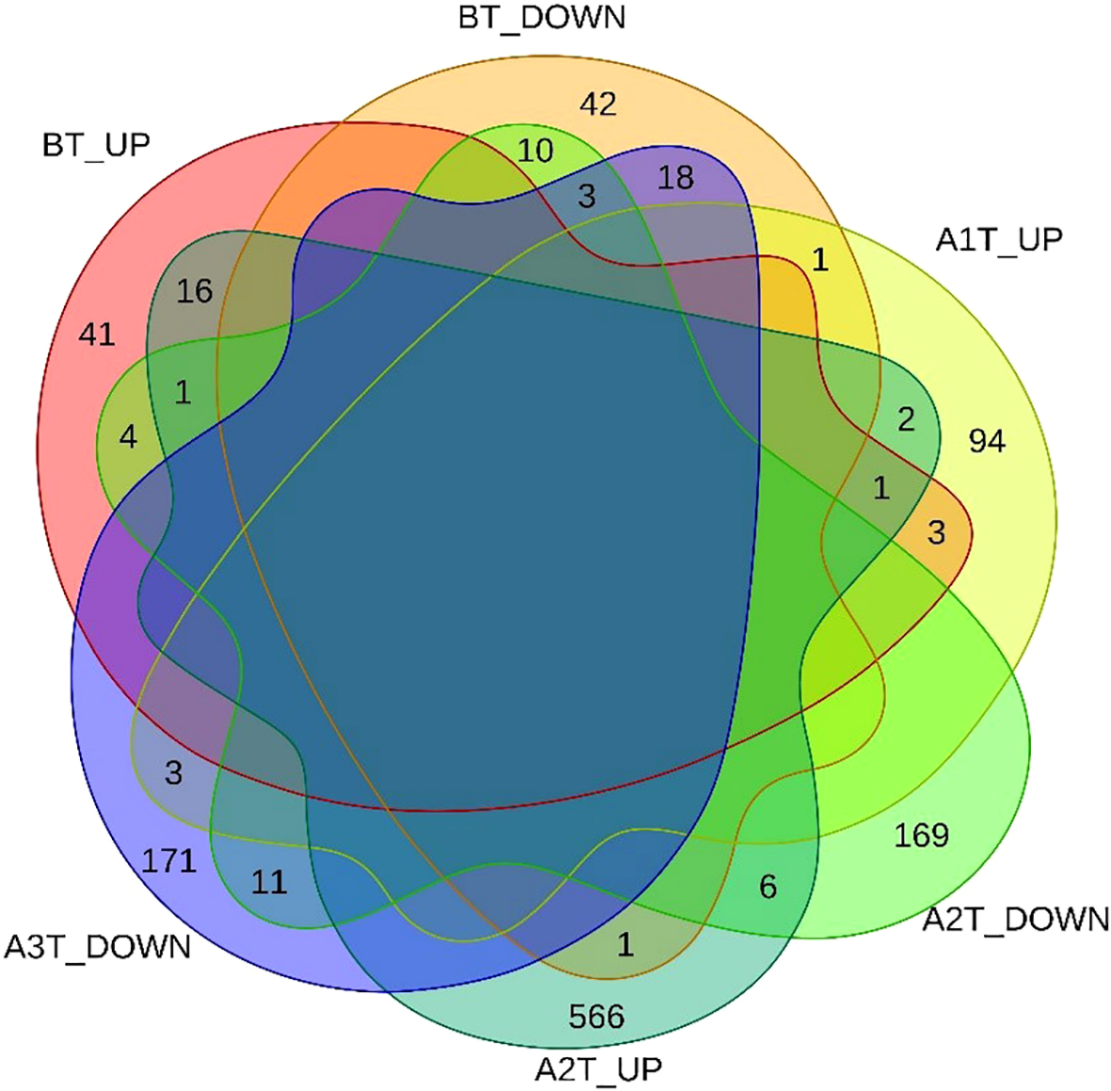
Venn diagram of DEGs across the three distinct stages: BT, A1T, A2T.

To assess the impact of sintilimab treatment on gene expression dynamics, we applied the LCGI method, identifying genes with consistent directional changes, either upregulated or downregulated, at both A1T and A2T stages, while rigorously excluding genes exhibiting similar BT expression changes. This approach yielded 13 DEGs, comprising two upregulated and 11 downregulated genes (**Figure 3**).

**Figure 4:**
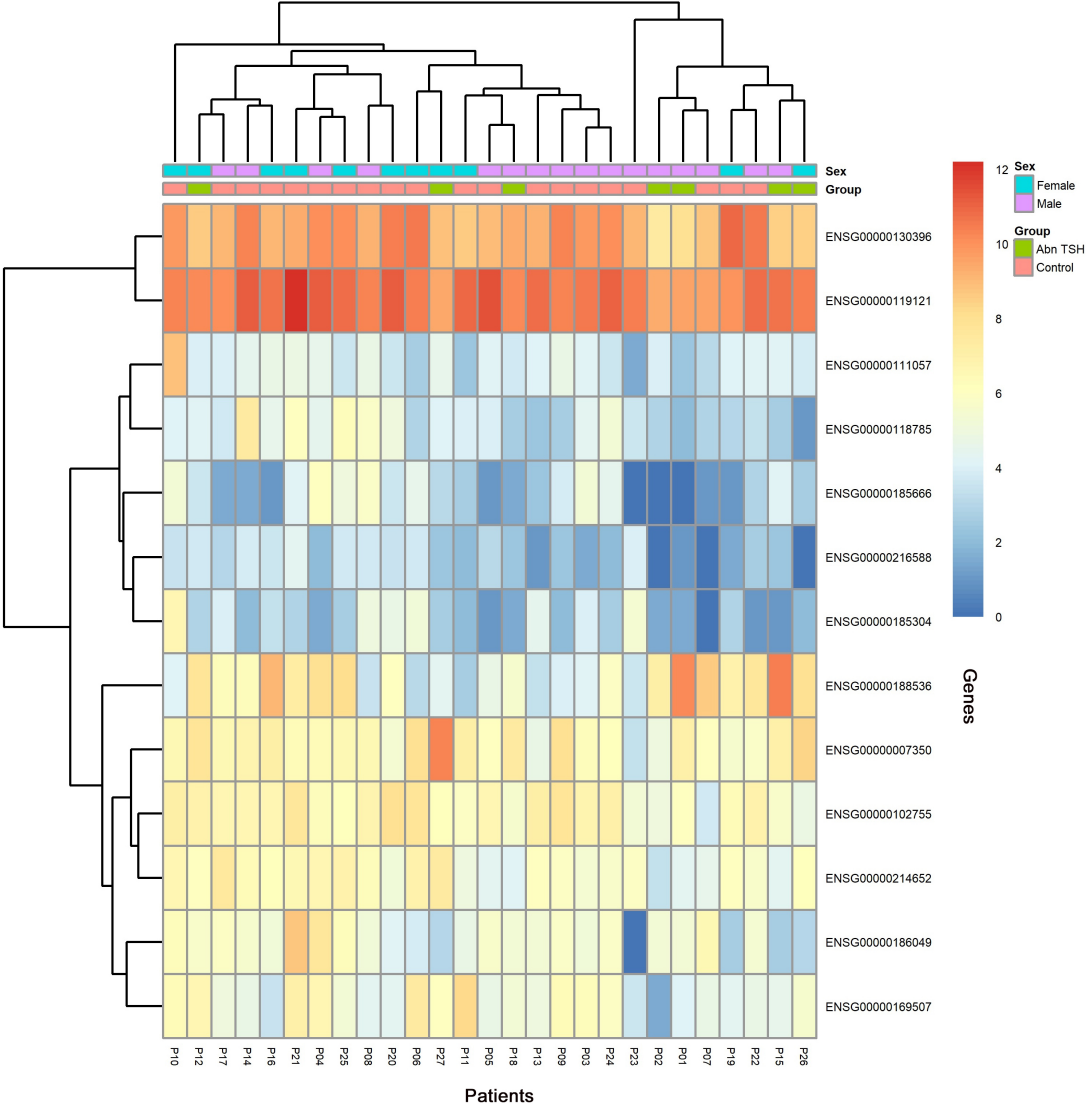
Heatmap of DEGs after the first treatment of sintilimab.

**Figure 5:**
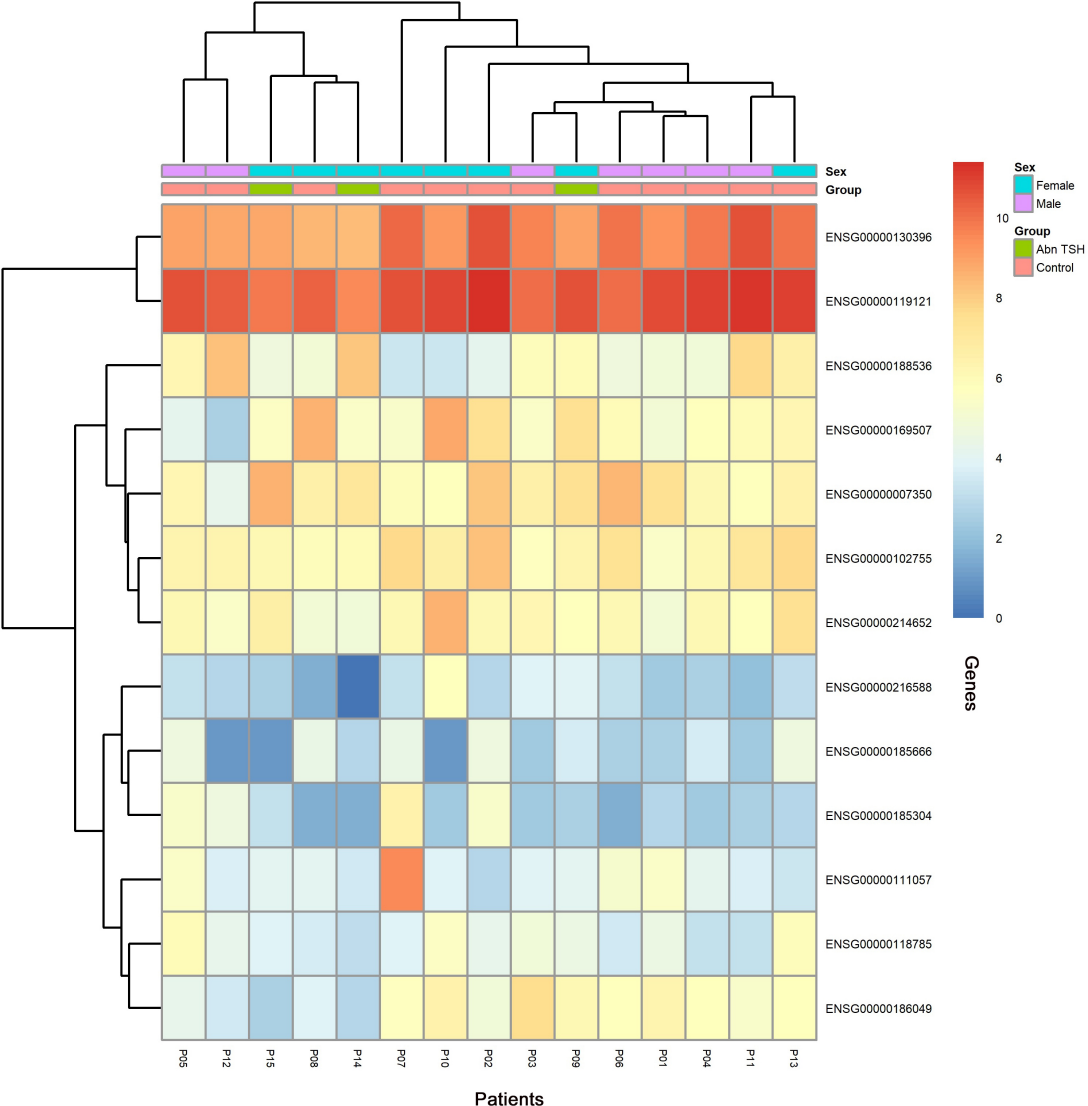
Heatmap of DEGs after the second treatment of sintilimab.

To capture progressive transcriptional adaptations, we retained a primary threshold of *p* < 0.05 and |log_2_ fold change| > 1 (highlighted as green dots on volcano plots) across all comparisons, while relaxing the fold change cutoff to |log_2_FC| > 0.5 specifically for the adjacent A2T phase to detect subtler expression shifts. Heatmaps (**Figures 4**, **5**) visualize critical gene expression patterns across samples post-first and second treatment cycles, with hierarchical clustering dendrograms at the top illustrating sample similarity.

### GO functional annotation and pathway enrichment analysis

**Figure 6:**
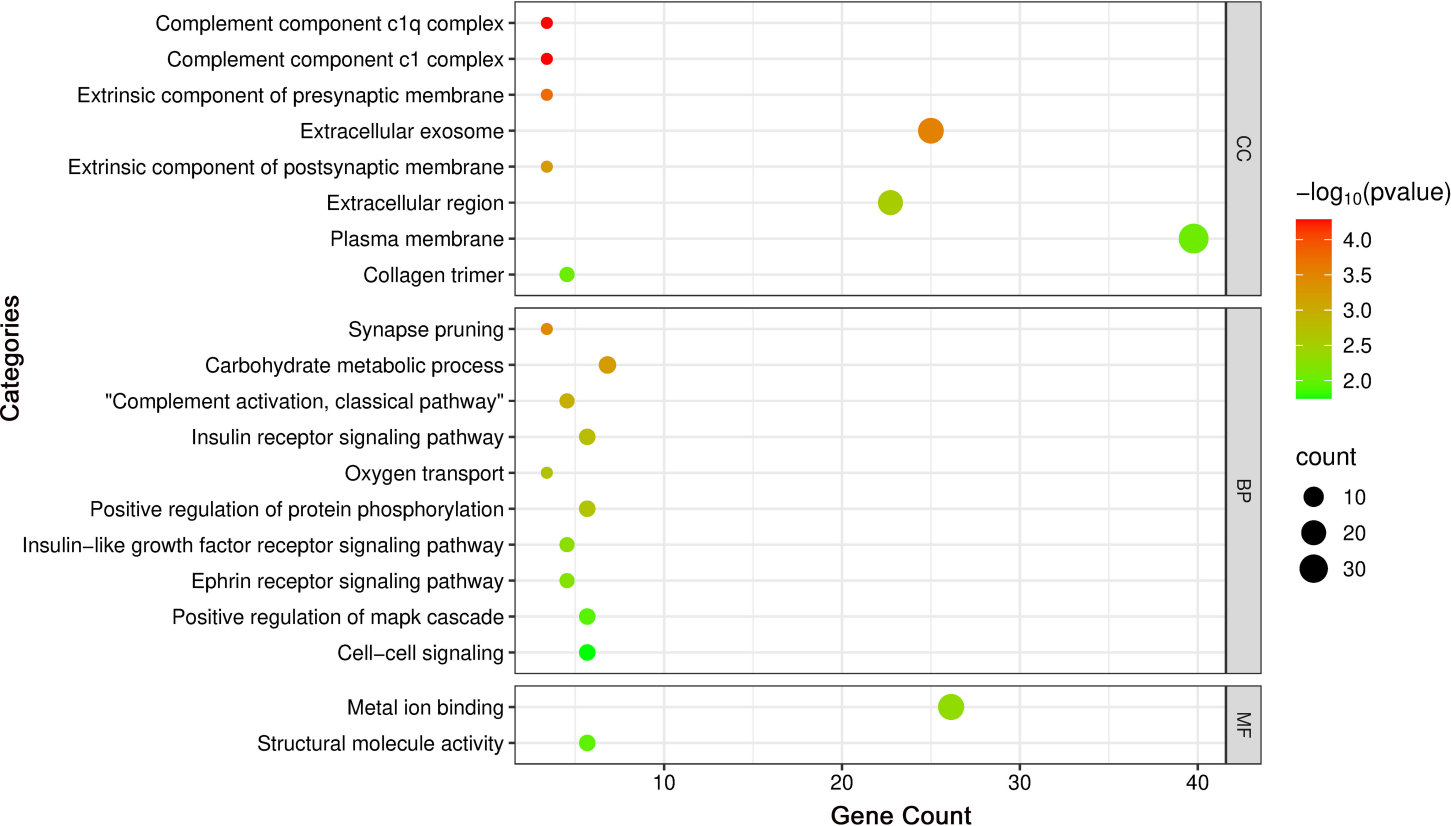
Bubble plot of GO enrichment analysis highlighting key terms.

GO enrichment results are visualized in the bubble plot (**Figure 6**). Key enriched terms, considering both enrichment ratio and *p*-value, converged on complement activation as a central biological process, implicating neuro-immune crosstalk alongside perturbations in metabolic and transport pathways that collectively contribute to thyroid irAEs.

**Figure 7:**
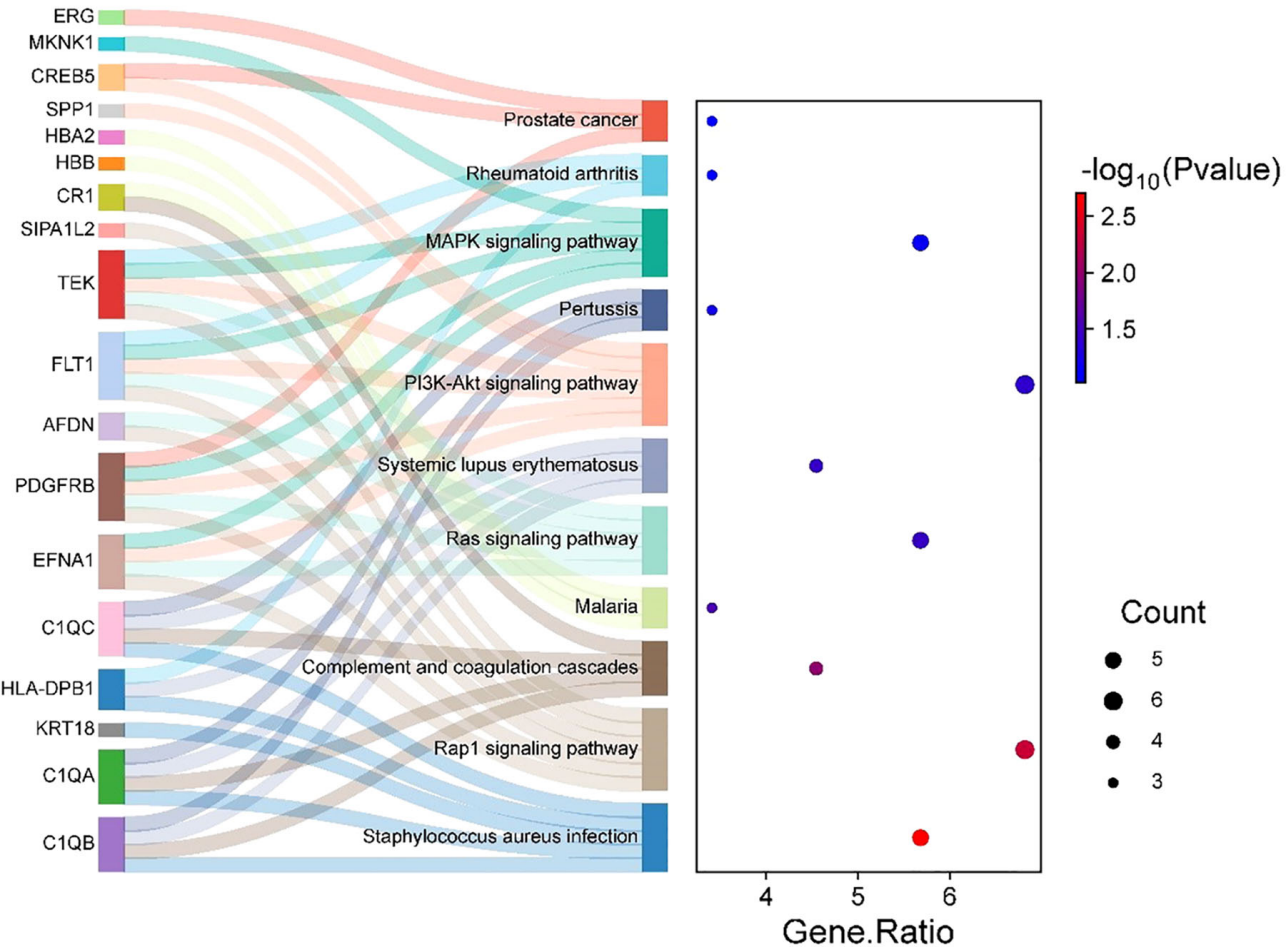
Sankey and bubble chart: gene-pathway associations.

**Figure 8:**
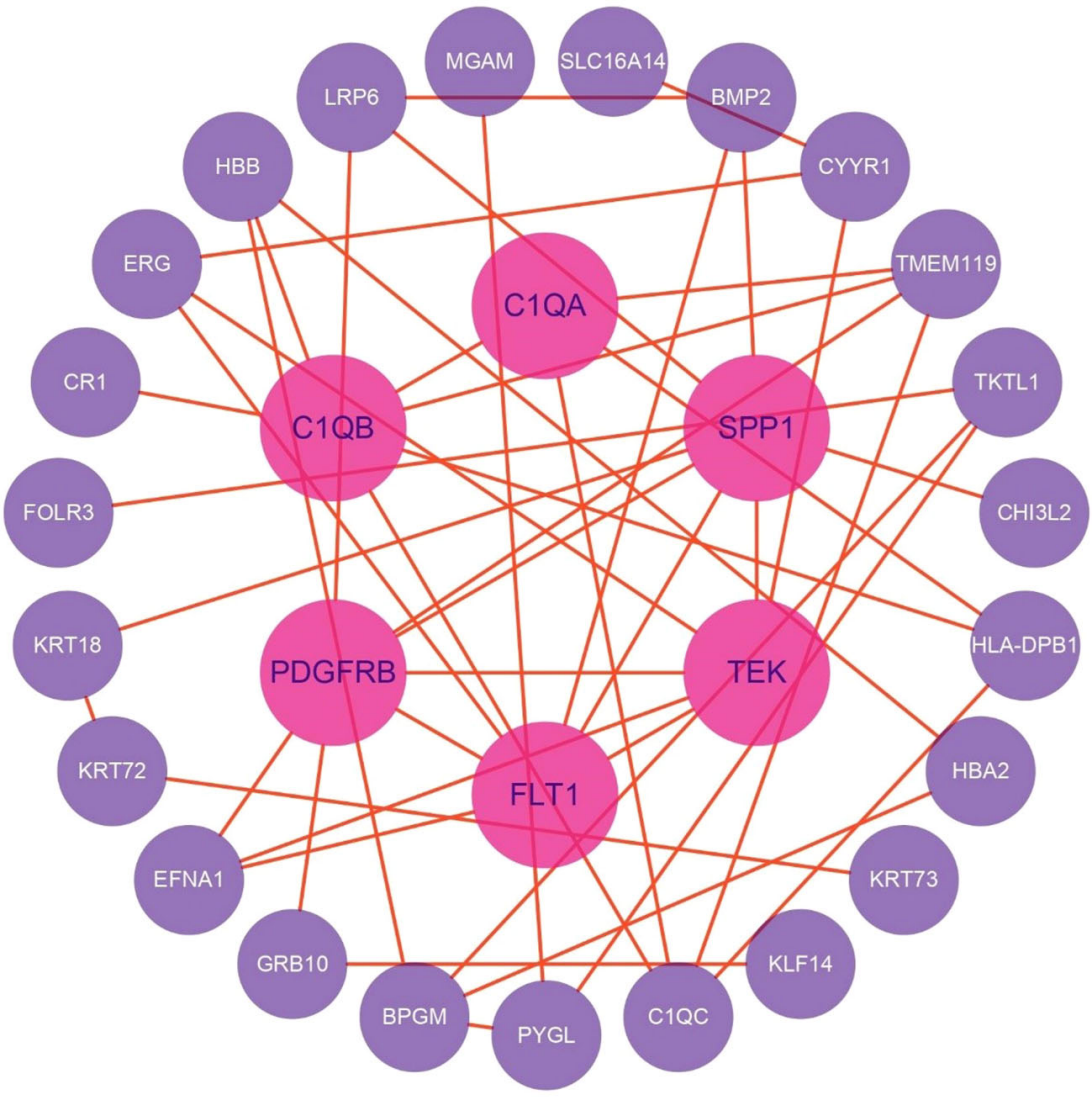
PPI network revealing key regulatory hubs in cellular pathways related to thyroid irAEs.

Subsequent KEGG pathway enrichment analysis, performed via the DAVID database, identified 18 DEGs enriched across multiple pathways. Eleven significantly enriched pathways were visualized using a bubble chart (**Figure 7**), and a Sankey diagram (**Figure 8**) was constructed to systematically map the gene-pathway relationships. These enrichment patterns emphasized the functional interplay between the complement and coagulation cascades (hsa04610) and the MAPK signaling pathway (hsa04010).

### Construction of PPI networks

DEGs were analyzed for PPIs using the STRING database, and the resulting network was visualized with Cytoscape software. After filtering out disconnected nodes based on network topology, the refined network comprised 29 nodes and 45 edges (**Figure 8**).

Key regulatory nodes were identified using the CytoHubba plugin with the MCC algorithm, highlighting genes with high connectivity or essential functional roles. Notably, PDGFRB, TEK, FLT1, C1QB, SPP1, C1QC, and C1QA emerged as top candidates (MCC ≥ 10).

Subnetwork detection via MCODE, using a K-core threshold of 2, revealed modules of densely interconnected nodes. The highest-scoring subnetwork included PDGFRB, EFNA1, C1QB, TMEM119, C1QA, TEK, HLA-DPB1, FLT1, and C1QC, connected by 16 edges. This module surpassed the degree cutoff threshold, designating it as the most functionally pivotal cluster within the PPI network.

### eQTL and integrative analysis

**Figure 9:**
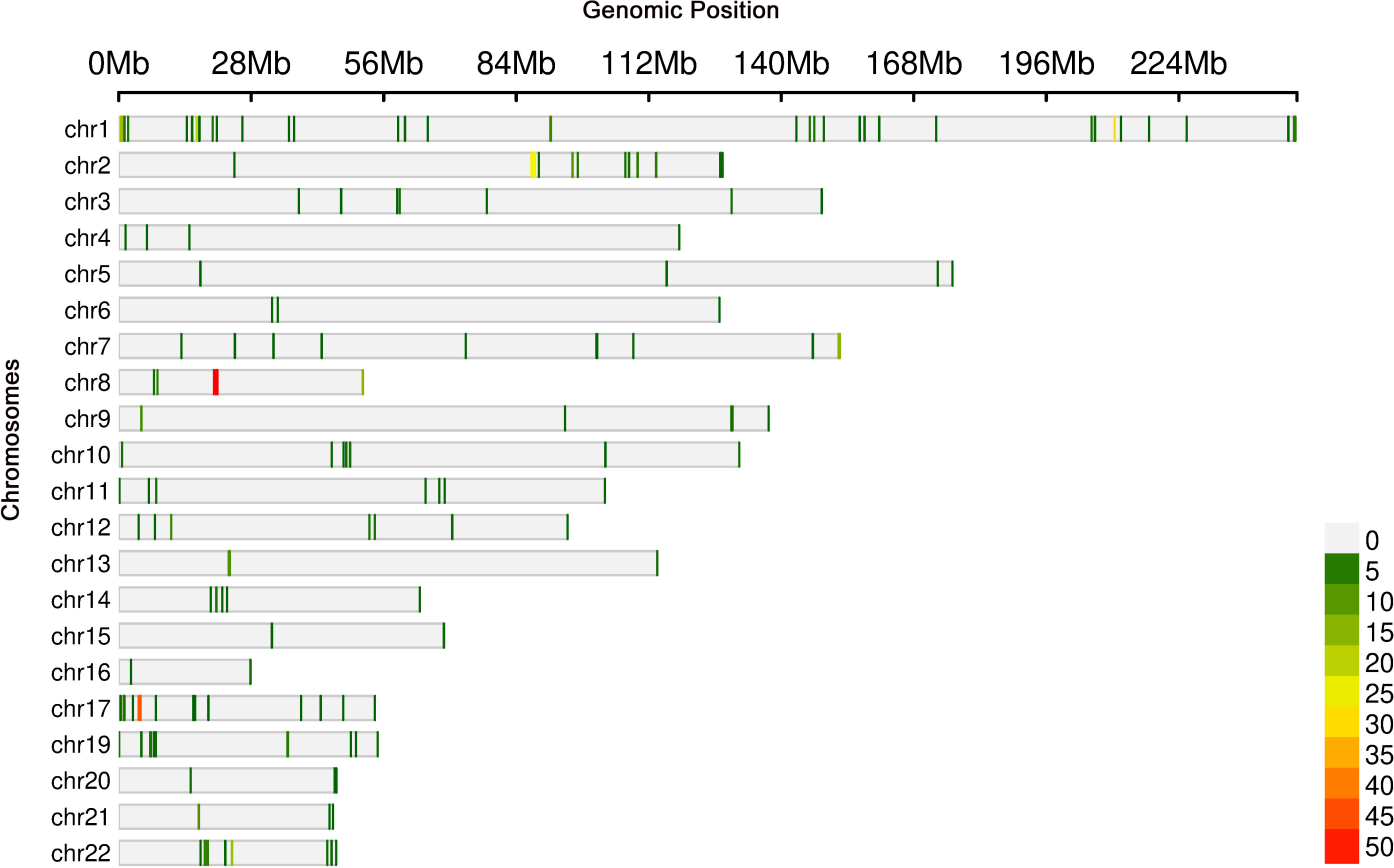
Genomic loci associated with gene expression levels identified through eQTL analysis.

Cis-eQTL analysis identified 514 significant associations involving 512 unique SNPs and 135 genes (**Figure 9**). Notably, the gene ADARB2 was found at the intersection of both the eQTL target gene set and DEGs. Three SNPs, rs12763675, rs2209625, and rs10904542, were implicated as putative eQTLs modulating ADARB2 expression, suggesting a potential role in the pathogenesis of thyroid irAEs.

Further integrative analysis using the STRING database revealed 173 distinct eQTLs regulating 37 genes that directly interact with DEGs. These 37 genes, in turn, influenced 39 downstream target genes, highlighting a complex regulatory network potentially contributing to thyroid irAE development.

## Discussion

The LCGI approach robustly filters out transient fluctuations by demanding consistent gene expression changes across multiple timepoints, thereby elevating confidence in detecting biologically meaningful and sustained signals. By requiring genes to exhibit concordant directional dynamics, either persistent upregulation or downregulation, across independent temporal measurements, LCGI effectively acts as an intrinsic experimental replicate. This design dramatically reduces the joint false-positive rate (<0.0025, derived from 0.05 × 0.05), markedly outperforming conventional single-timepoint FDR-controlled analyses, a feature especially advantageous in studies constrained by limited sample sizes.

Importantly, given that irAEs reflect progressive disturbances in immune homeostasis, such as sustained T-cell activation and cumulative inflammatory cytokine release, LCGI selectively enriches for genes with durable transcriptional responses intimately linked to irAE pathogenesis, while efficiently excluding ephemeral stress-induced gene expression. This targeted focus on stable molecular signatures not only enhances mechanistic insight but also improves the identification of robust biomarkers predictive of irAE onset.

Among the 13 genes identified via LCGI, several emerged as central hub nodes within the PPI network and have been experimentally validated for their pivotal roles in immune regulation. Notably, SPP1 (secreted phosphoprotein 1, also known as Osteopontin) orchestrates both innate and adaptive immune responses by modulating immune cell activation, proliferation, and differentiation, thereby finely tuning the magnitude of immune reactions (21). Through interactions with integrins (e.g., αvβ3) and the CD44 receptor, SPP1 facilitates immune cell trafficking to sites of inflammation (22, 23). Its chemotactic properties likely promote T-cell migration into healthy tissues, potentially driving the pathogenesis of irAEs.

Functionally, SPP1 modulates the secretion of key pro-inflammatory cytokines, including IL-1β, TNF-α, and IL-6, while concurrently dampening cytotoxic CD8^+^ T cell activity (23). Prior studies have demonstrated a strong positive correlation between SPP1 expression and the infiltration of diverse immune cell subsets in cancer, implicating it in the establishment of an immunosuppressive tumor microenvironment (24, 25). Moreover, genetic variants of SPP1 have been significantly linked to susceptibility and clinical severity in autoimmune diseases such as systemic lupus erythematosus (SLE) and rheumatoid arthritis (RA), underscoring its potential as a biomarker for irAEs (21).

Within the PPI network, C1QA, C1QB, and C1QC formed a prominent subnetwork identified through CytoHubba and MCODE analyses, implicating their involvement in thyroid irAEs. C1q, a hexameric protein complex composed of these three distinct subunits, plays a central role in maintaining immune homeostasis by mediating immune complex clearance, regulating phagocytosis, balancing cytokine production, and modulating T-cell subsets. Dysfunction of C1q has been directly linked to the onset and progression of autoimmune diseases such as SLE and RA, underscoring its crucial function in immune tolerance and disease susceptibility (26).

Elevated C1q expression correlates strongly with disease activity and inflammatory severity in autoimmune conditions, including RA (27) and Takayasu arteritis (TA). Moreover, fluctuations in C1q levels have emerged as a promising biomarker for monitoring the therapeutic efficacy of immunosuppressive treatments (28). Notably, during the A1T stage, transcripts of C1QA, C1QB, and C1QC were significantly upregulated relative to controls, suggesting that immune hyperactivation following sintilimab administration might increase susceptibility to thyroid irAEs.

These observations illuminated the dual role of C1q in autoimmune pathophysiology and treatment response, emphasizing the necessity for careful endocrine irAE monitoring in patients undergoing ICI therapy.

Based on the constructed PPI network and the outcomes of GO and KEGG enrichment analyses, we proposed a pathogenic cascade underpinning thyroid irAEs, driven by coordinated gene functional disruptions. Downregulation of FLT1 undermines vascular endothelial stability (29), which, in concert with decreased TEK expression, perturbs ANGPT/TIE2 signaling homeostasis. This destabilizes VE-cadherin-mediated adherens junctions, compromising endothelial barrier integrity and markedly increasing vascular permeability (30). Simultaneously, upregulation of PDGFRB exacerbates barrier dysfunction, facilitating vascular leakage and inflammatory cell infiltration.

Concurrently, elevated expression of C1QA, C1QB, and C1QC activates the classical complement cascade, promoting formation of C3 convertase (31) and membrane attack complexes (MAC) that directly induce lysis of thyroid follicular cells (32). Downregulation of keratins further destabilizes the cellular cytoskeleton (33), synergizing with complement-mediated damage to release autoantigens such as thyroglobulin (Tg) and thyroid peroxidase (TPO).

**Figure 10:**
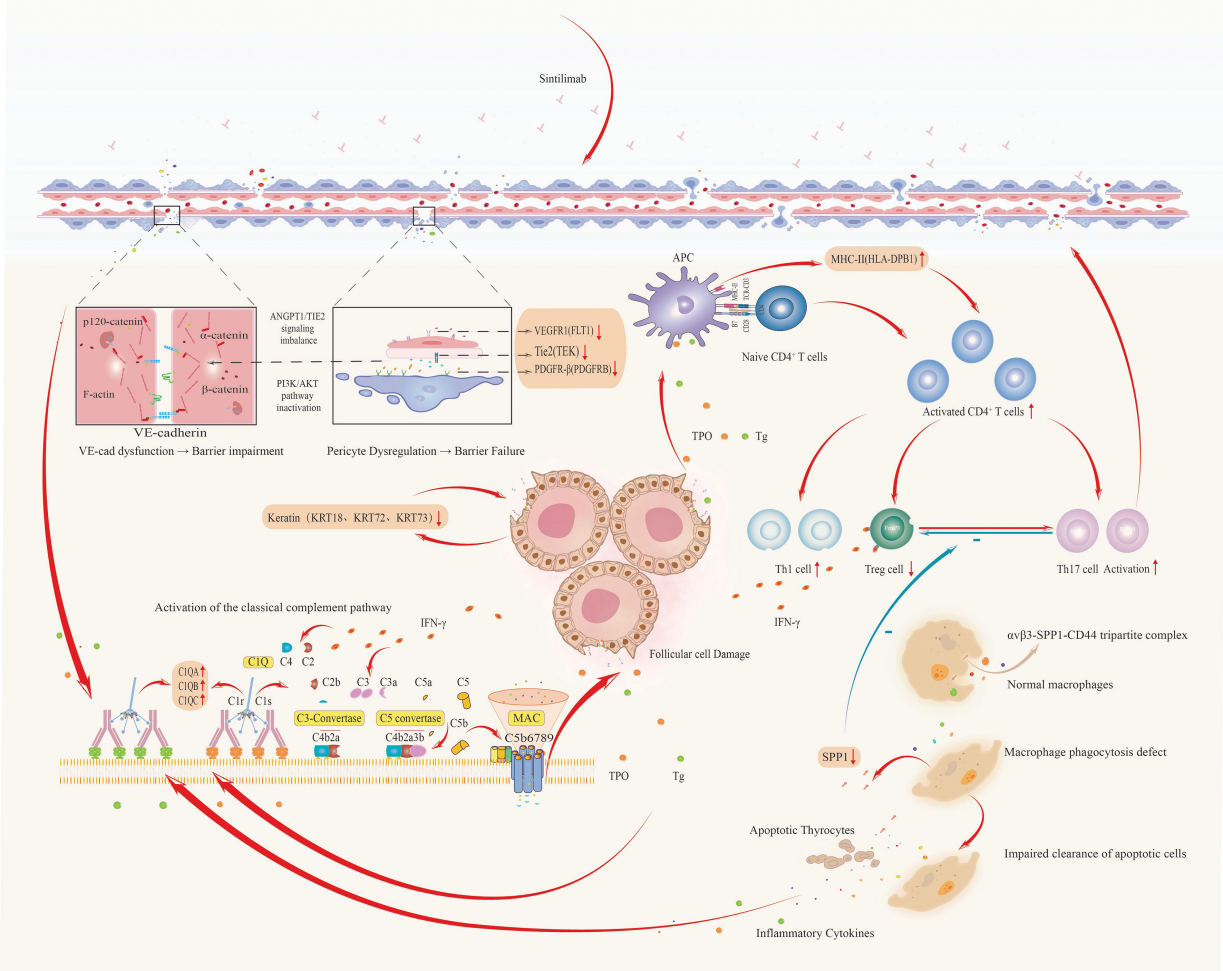
Integrated pathogenic cascade of thyroid irAEs: from vascular dysfunction to autoimmune amplification.

Reduced SPP1 expression impairs macrophage-mediated clearance of apoptotic debris (34), intensifying antigen exposure, while disrupting the Treg/Th17 balance (35), thereby diminishing immunosuppressive control and amplifying pro-inflammatory responses. The resulting autoantigens are efficiently presented by antigen-presenting cells with upregulated HLA-DPB1, triggering activation of autoreactive CD4^+^ T cells. These T cells secrete interferon-gamma (IFN-γ), which further upregulates HLA-DPB1 expression (36) and complement production, establishing a self-perpetuating feedback loop of antigen presentation and inflammatory amplification. This cascade culminates in relentless thyroid autoimmune injury (**Figure 10**).

Leveraging STRING-based analysis of eQTL-targeted genes, we identified 12 genes that directly interacted with 11 candidate genes (FLT1, PDGFRB, TEK, C1QA, C1QB, C1QC, HLA-DPB1, SPP1, KRT72, KRT73, KRT18) implicated in thyroid irAE pathogenesis. These 12 genes were linked to 75 SNPs. Notably, C3 emerged as a central bridging gene, engaging directly with four key mechanistic genes, SPP1, C1QA, C1QB, and C1QC, while genetically associated with three specific SNPs (rs189966229, rs9749508, and rs148292769). This revealed a novel genetic-immunological axis centered on C3-associated eQTLs, orchestrating complement-mediated tissue injury as a core driver of thyroid irAEs.

The identified SNPs modulated C3 expression, positioning it as a pivotal node that translated genetic susceptibility into downstream effector pathways. Through its interactions with SPP1 and the complement initiators C1QA/B/C, C3 formed a “complement-matrix axis” that promoted macrophage polarization toward a pro-inflammatory phenotype, thereby amplifying pathogenic immune responses.

## Conclusion

In summary, our longitudinal transcriptomic investigation delineated dynamic gene expression profiles associated with thyroid irAEs during sintilimab therapy. By integrating genomic variants with transcriptomic data via eQTL analysis, we constructed a comprehensive genetic framework underlying thyroid irAEs, illuminating the intricate links between genetic variation and clinical phenotypes. Notably, this study is the first to identify C3 eQTLs as a central regulatory nexus bridging genetic susceptibility and complement-driven thyroid irAEs. Therapeutic targeting of this pathway holds promise for dissociating antitumor efficacy from autoimmune toxicity, offering a refined strategy for precision immunotherapy.

Despite these scientifically and clinically impactful findings, limitations remain. Restricted by sample size and follow-up duration, validation in larger, longitudinal clinical cohorts is essential. Furthermore, mechanistic insights into SNP functionality require dedicated *in vitro* and *in vivo* studies to unravel the causal pathways linking genetic variation to irAE pathogenesis.

## Data Availability

The raw data supporting the conclusions of this article will be made available by the the corresponding authors.
